# Synthesis of multi-band reflective polarizing metasurfaces using a generative adversarial network

**DOI:** 10.1038/s41598-022-20851-y

**Published:** 2022-10-11

**Authors:** Parinaz Naseri, George Goussetis, Nelson J. G. Fonseca, Sean V. Hum

**Affiliations:** 1 The Edward S. Rogers Sr. Department of Electrical & Computer Engineering, University of Toronto, Toronto, Canada; 2 Institute of Sensors Signals and Systems, Heriot-Watt University, Edinburgh, Scotland; 3 Antenna and Sub-Millimetre Waves Section, European Space Agency (ESA), Noordwijk, The Netherlands

**Keywords:** Electrical and electronic engineering, Applied optics, Scientific data

## Abstract

Electromagnetic linear-to-circular polarization converters with wide- and multi-band capabilities can simplify antenna systems where circular polarization is required. Multi-band solutions are attractive in satellite communication systems, which commonly have the additional requirement that the sense of polarization is reversed between adjacent bands. However, the design of these structures using conventional ad hoc methods relies heavily on empirical methods. Here, we employ a data-driven approach integrated with a generative adversarial network to explore the design space of the polarizer meta-atom thoroughly. Dual-band and triple-band reflective polarizers with stable performance over incident angles up to and including 30°, corresponding to typical reflector antenna system requirements, are synthesized using the proposed method. The feasibility and performance of the designed polarizer is validated through measurements of a fabricated prototype.

## Introduction

Electromagnetic (EM) metasurfaces are two-dimensional versions of metamaterials with a thin profile. These surfaces, which are composed of sub-wavelength meta-atoms that are judiciously engineered from dielectric and/or metallic scatterers, allow for the manipulation of the amplitude and phase of the impinging electromagnetic waves based on frequency, polarization, and incident angle. This extraordinary ability to achieve wave manipulation has enabled metasurfaces to address needs in numerous potential applications for future generations of imaging, quantum optics, and wireless systems^1,2^.

**Figure 1:**
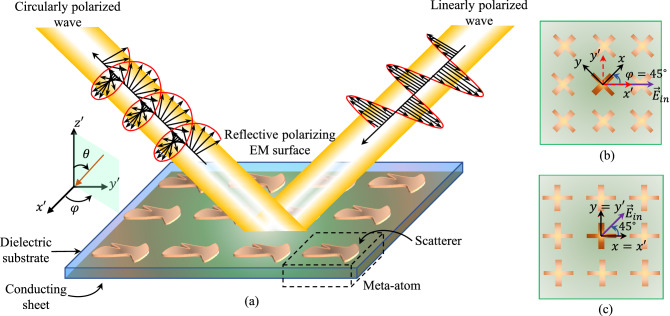
(**a**) A reflective polarizer shown with the relation between the coordinate systems of the polarizering scatterer, (*x*, *y*), and the uniform array $(x',y')$ when: (**b**) (*x*, *y*) is rotated $\varphi =45^\circ$ compared to $(x',y')$, where $\varphi$ is the angle from the $x'$-axis in $x'y'$-plane, and (**c**) (*x*, *y*) and $(x',y')$ are the same.

EM metasurfaces that convert linear polarization (LP) to circular polarization (CP) and vice versa, which we will simply refer to as polarizers, have attracted significant interest, especially in the microwave regime^3–16^. This is because circular polarization is more effective than linear polarization for establishing and maintaining mobile communication links due to their robustness to channel-induced effects from absorption, cross-polarization interference, impaired line-of-sight paths, Faraday rotation effect due to the ionosphere, and multi-path propagation. Polarizers can operate in reflection^3–9,17^ or transmission mode^10–16^, where the incident linearly-polarized wave is converted to a reflected or transmitted circularly-polarized wave, respectively. The transmissive polarizers are great solutions for integration with linearly-polarized antennas; however, they are usually composed of more than one layer of scatterers and can introduce insertion loss to the converted wave. Among these surfaces, reflective single-layer polarizing metasurfaces, shown in Fig. 1, operating in multiple frequency bands, are desirable due to the simplicity of their fabrication process and low losses. In satellite communications for a single-feed-per-beam (SFB) configuration, dual-band polarizers allow to reduce the number of the radiating apertures while enabling spectrum reuse, resulting in considerable savings in payload mass and volume^4,5,9–11^. In these applications, the polarizer is spatially fed by a nearby radiating antenna (feed), resulting in the incident wave arriving at the polarizer at different angles. Moreover, for multibeam generation where multiple feeds are illuminating the surface of the polarizer, the incident angle at each meta-atom from each feed can be different. This creates the requirement for performance that is stable with the angle of incidence. Due to ever-growing need for wider frequency bands and higher data rates in wireless systems, there is significant interest in not only improving the performance of dual-band polarizers in terms of bandwidth and oblique incidence performance, but also developing multi-band polarizing metasurfaces given the relative scarcity of such designs.

Communication satellites generally implement frequency division duplexing to avoid overwhelming receivers due to the extreme difference of power between the transmitted and the received signals. To further isolate the signals, orthogonal polarizations are used between the uplink and the downlink bands, providing an additional design constraint for reflective polarizing surfaces. Therefore, the polarizer must operate not only in non-adjacent frequency bands, but also exhibit selectivity based on the polarization sense of the incident wave in a certain frequency band. To synthesize an electromagnetic polarizer, a designer needs to make important choices for the meta-atom’s scatterer shape, period, substrate thickness, and dielectric permittivity. Moreover, it is desired that the polarization conversion in all frequency bands be achieved in line with the polarization plan, with low axial ratio under both normal and oblique incidence over broad frequency ranges. A metasurface-based polarizer can offer these diverse and simultaneous properties through their constituent sub-wavelength patterned metallic and/or dielectric scatterers. There have been some attempts to implement a systematic design process using equivalent circuit models^4,9,12,14,18^. Nevertheless, the solution space of the meta-atom shape is an important factor that can be further leveraged in improved designs. It has been shown that designs with more complicated scatterer shapes that are not directly derived from an equivalent circuit model can have more compact profile and improved performance^5,19^. In another systematic approach, the scatterer shape has been treated as a pixelated structure and genetic algorithms or other types of optimization methods have been employed to find the global optimum^20^. This method unnecessarily increases the dimensions of the design space, which makes the optimization problem more challenging. On the other hand, in an empirical approach, a designer runs many iterations of simulations based on experience to find the right choice for the meta-atom’s scatterer shape and dimensions to meet all the requirements. However, even in the cases that this approach yields a successful design, it is time-consuming, resource-demanding, and might lead to designs far from the global optimum. Therefore, this creates the need for an automated and effective way to explore the true potential of the scatterer design space.

Data-driven machine learning (ML) methods have revolutionized the discovery of new materials with desired and novel properties in the chemical, pharmaceutical sciences^21–24^, and wave-matter interactions^25–38^. In particular, generative ML methods such as generative adversarial networks (GANs)^39,40^ and variational autoencoders (VAEs)^41^ have received significant attention recently for exploring the possible solution space. In such inverse problems, ML techniques can exploit the key features hidden in training samples and generate new samples with improved properties. While both networks are capable of synthesizing new and unique outputs after training, VAEs rely on interpolating the features of the training samples whereas GANs can create more unique and diverse shapes. This is due to the difference between the latent spaces, which represent the features of the training dataset, constructed by a VAE or a GAN. The latent space of a VAE is continuous and clustered by design because of the Kullback–Leibler (KL) divergence^41^ and reconstruction losses in the training loss function, which makes it suitable for interpolation and optimization. This also makes the latent space constructed by a VAE suitable as an input to surrogate models to predict the properties of a dataset^21,42^. However, since minimizing the reconstruction loss or KL divergence is not the focus in training of a GAN, the latent space can include more diverse features.

Since the rise of ML methods, there have been many efforts to apply them to the design of the EM structures^42–58^. Some employ forward neural networks as surrogate models to predict the properties of meta-atoms composed of scatterers with canonical shapes and accelerate the search in the design space for the optimized meta-atom^43–47^. Others develop inverse neural networks^48,49^ to predict the shape of the scatterer based on the desired properties. More recently, others have taken advantage of the generative networks to explore the design space further^42,50–58^. It is worth noting that ML design frameworks for optical metasurfaces can benefit from the relation between the dielectric scatter shape and the refractive index to find the optimum design through a global optimizer^59^. However, the lack of such a relation in metasurfaces composed of metallic scatterers makes it more challenging. Moreover, frameworks that output scatterers with isolated metallic pixels require a significant amount of simulations due to the unnecessarily enlarged design space.

Here, for the first time, the design of a polarizer has been treated as the synthesis of a new material with desired electromagnetic (EM) properties by employing a GAN. The unique and difficult aspect of the inverse design of a multi-band polarizer is that unlike resonant metasurfaces^53,54^, the desired scattering parameters cannot be explicitly and simply defined using resonances. In fact, there can be many valid solutions that present the desired axial ratio but have different resonant linearly-polarized scattering parameters. Hence, it is essential for the proposed approach to tackle the inverse design of a polarizer using high-level frequency dispersive criteria on the axial ratio. In fact, such a multi-band design approach goes beyond conventional ones for this type of structure, since a deterministic approach that simultaneously associates the geometry with the scattering behavior across two or three frequency bands is not available. Moreover, while interpolation of scatterer shapes to achieve the required phase and amplitude electromagnetic responses in an efficient and expedited manner is expected from generative machine learning models^42,50–58^, the task at hand here is more challenging due to the scarcity of existing polarizers with the aforementioned acceptable combination of electromagnetic properties. Here, we aim to test if a GAN could generalize/extrapolate from a relatively small data set to derive a sophisticated, dipersion-optimized design.

Here, the proposed GAN-based approach is employed to first design a single-layer reflective dual-band polarizer that reflects a given incident LP wave (e.g. horizontal polarization) to different CP senses in the adjacent bands, e.g. left-handed CP (LHCP) in one band and right-handed CP (RHCP) in the other band. In the SFB configuration proposed by Fonseca et al.^4^, such polarizer produces the orthogonal uplink and downlink CP beams in adjacent spot beams by LP feeds located at different physical locations. This avoids the physical overlap between the horizontal and vertical LP feeds, resulting in a significantly simpler antenna system. Furthermore, the polarizer needs to maintain these properties for wide axial ratio bandwidths in the desired frequency bands under both normal and oblique incidence. After the successful design of the dual-band polarizer, we demonstrate that the same dataset and trained GAN can be employed to propose a new triple-band polarizer with wide bandwidths performance and orthogonal CP polarizations under both normal and oblique incidence.

## Reflective polarizering metasurfaces

A metasurface-based polarizer is a uniform array of meta-atoms composed of one (or more) layer(s) of dielectric substrates and/or metallic scatterers, shown in Fig. 1a. The orientation of the scatterer in this array can be often any of the two cases of Fig. 1b and c. One coordinate system can be defined for the array, $(x',y')$, where the two axes are aligned with the sides of the array when another coordinate system can be defined for the scatterer, (*x*, *y*). The orientation of the (*x*, *y*) coordinate system is chosen such that it is usually aligned with scatterer features that result in maximum co-polarization reflection, $\Gamma _{xx}$, $\Gamma _{yy}$, and minimum cross-polarization reflection, $\Gamma _{xy}$, $\Gamma _{yx}$. This means that if the scatterer is excited by an *x* (or *y*)-polarized wave, the reflected wave in the *y*-direction (or *x*-direction) would be negligible. In case of Fig. 1b, $(x',y')$ is rotated $45^\circ$ compared to (*x*, *y*) whereas in case of Fig. 1c, the array’s and the scatterer’s coordinate systems are aligned.

A reflective polarizer is designed such that the incident linearly polarized wave can be decomposed into two equal LP components along the axes of its coordinate systems, *x* and *y*. Then, it reflects both of them with unit amplitude and a differential phase between them of $(2i+1)\frac{\pi }{2}$, where *i* is an integer. That way, the reflected wave is circularly polarized. In order for this to happen, the incident field has to be linearly polarized along either $45^\circ$ or $135^\circ$ rotated compared to the coordinate system of the scatterer, (*x*, *y*), shown in Fig. 1b and c. Therefore, in case of Fig. 1b, the polarizering surface is excited by a $\varphi =0^\circ$ or $90^\circ$-directed LP wave to obtain a CP reflected wave, where $\varphi$ is the angle from the $x'$-axis in $x'y'$-plane. Conversely, in case of Fig. 1c, the polarizering surface is excited by a $\varphi =45^\circ$ or $135^\circ$-slanted LP wave to obtain a reflected CP wave.

Without loss of generality, we focus on the design of a passive polarizer in the configuration of Fig. 1c. As mentioned earlier, in this type of polarizer, the polarization of the incoming wave is rotated such that $\varphi =45^\circ$ or $135^\circ$ compared to the scatterer’s and array’s coordinate systems. To generate this incident wave, the polarizer is excited by both *x*- and *y*-directed electric fields simultaneously. For an ideal reflective passive polarizer, all the fields are reflected with no loss. Then, the performance of the polarizer can be evaluated by the axial ratio (AR) of the reflected circularly polarized wave when the polarizer is excited by a linearly polarized wave. Once it is established that the cross-polarization reflection coefficients are negligible, which depends on the shape of the scatterer, we define the AR in dB as

1 $$AR =\text {sgn}(\sin \delta _{ph}){20\log _{10}}\sqrt{\frac{|{\Gamma _{xx}}|^2+{|{\Gamma _{yy}}|}^{2}+|{{\Gamma _{{xx}}}^{2}}+{{\Gamma _{{yy}}}^{2}}|}{{|{\Gamma _{xx}}|}^{2}+{|{{\Gamma _{yy}}}|}^{2}-|{{\Gamma _{{xx}}}^{2}}+{{\Gamma _{{yy}}^{2}}|}}},$$

where $\delta _{ph}= \arg (\Gamma _{xx})- \arg (\Gamma _{yy})$. Here, the sign of the AR indicates the handedness of the CP wave. A positive AR in dB indicates the CP wave is right-handed, whereas a negative dB value indicates the CP wave is left-handed. The choice of sign here is arbitrary.

Typical performance requirements call for the absolute value of the AR of the reflected CP wave to be less than 3.0 dB in the band(s) of interest. Moreover, for satellite communication applications, a polarizing reflector is spatially fed by a feed antenna that is placed at the distance *F* from the reflector, typically corresponding to the focal length of a paraboloid, with a projected aperture of diameter *D*. For $F/D>1$, which is typical in these configurations, constituent meta-atoms of the polarizer receive the incoming wave under either normal or oblique incidence up to and including $\theta =30^\circ$, shown in Fig. 1. Therefore, it is important that the meta-atoms have stable performance for both normal and oblique incidence. This means that the absolute value of the AR of the reflected CP wave from all the meta-atoms across the surface should be below 3.0 dB in the band(s) of interest. An additional requirement for a multi-band polarizer for satellite communications is that the sense of reflected CP waves is reversed between the operation frequency bands. These requirements can be summarized as acceptable minimum ($AR_{min}$) and maximum levels for the AR ($AR_{max}$) versus frequency. An error evaluating how much the meta-atom’s AR is within the indicated bounds is defined as

2a $$\begin{aligned} e_{AR}=\Vert (AR(f)-AR_{min}(f))(AR(f)-AR_{max}(f)) + |(AR(f)-AR_{min}(f))((AR(f)-AR_{max}(f))|\Vert \end{aligned}$$

2b $$\begin{aligned} ={\left\{ \begin{array}{ll} 0 &{} \text {if}\quad AR_{min}(f) \le AR(f) \le AR_{max}(f) \quad \forall f \\ 2|(AR(f)-AR_{min}(f))((AR(f)-AR_{max}(f))| &{} \text {otherwise} \end{array}\right. } \end{aligned}$$

where *f* indicates the frequency in the bands of interest. It is worth noting that $e_{AR}$ tends to zero if *AR*(*f*) is within the bounds defined by $AR_{min}$ and $AR_{max}$ in all bands of interest. Next, we show how we use $e_{AR}$ to not only find the optimized polarizing meta-atom, but also to guide the machine-learning generator by providing it with examples of “*good*” polarizers.

## Using a GAN to design a polarizer

A generative adversarial network is a combination of two neural networks: a *generator* and a *discriminator*. The generator produces samples given a random noise vector. The discriminator is provided with a training data based on which it predicts if the produced samples by the generator are “authentic” or not. The two networks are in contest with each other. This means that during the training process, the discriminator tries to accurately predict the probability of the samples from the training data as 1 while predicting the probability of the samples produced by the generator as 0. Meanwhile, the generator tries to create samples that have similar features to the ones of the training data samples and convince the discriminator to predict a probability value close to 1 for the generated samples. Therefore, once the training process is completed, the generator learns the features of the training data samples and can be employed to explore and expand a solution space that is defined by the training data. GANs have been successfully used in image processing field to create convincing images of humans, animals, and objects^60–62^.

At each training epoch, the discriminator is fed a batch of inputs for which it predicts probability values. Half of this batch is *real*, randomly chosen from the training data (*x*). The other half is created by the generator given random noise vectors, *z*, with size *m*. The elements of the noise vector are sampled from a normal distribution *N*(0, 1). The GAN was originally optimized by the cross-entropy between the distributions of the real and generated data. Basically, the discriminator tries to maximize while the generator tries to minimize the following value^39^

3 $$\begin{aligned} E_x[\log (D(x))]+E_z[\log (1-D(G(z))]. \end{aligned}$$

In Eq. (3), *G*(*z*) is the generator’s output provided the noise vector *z*; *D*(*x*) and *D*(*G*(*z*) are the predicted probabilities by the discriminator of the real data *x* and generated data *G*(*z*), respectively. $E_x$ and $E_z$ are the expected values over all the real and generated data, respectively. It was noted that at the beginning of the training process when it is easy for the discriminator to distinguish between the real and generated inputs, optimizing the value in Eq. (3) prevents the GAN from being effectively trained^39^. Alternatively, the gradients of the binary cross-entropy loss function

4 $$\begin{aligned} L = -\frac{1}{N} \sum _{k=1}^{N} y_k\log [p(y_k)]+(1-y_k)\log [1-p(y_k)], \end{aligned}$$

where $y_k$ and $p(y_k)$ are the label and the predicted probability by the discriminator for an input, respectively, are used to update the discriminator and the generator separately. When the discriminator is to be updated, the combined batch of real (labeled with 1) and generated data (labeled with 0) are used as inputs as described above. Conversely, when the generator is updated the whole batch of inputs is produced by the generator but labeled with 1. The latter effectively minimizes $[\log (1-D(G(z))]$ or maximizes $[\log (D(G(z))]$, which is the only part in Eq. (3) that the generator can control.

**Figure 2:**
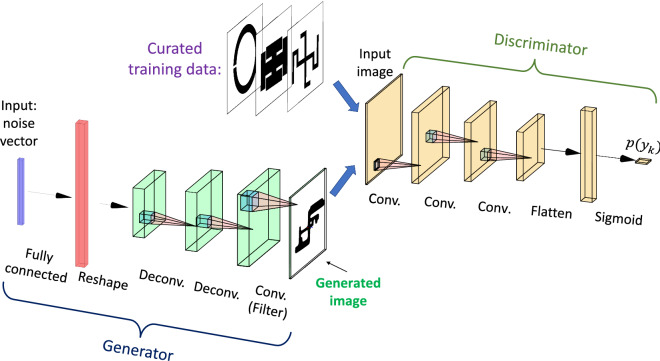
Representative architecture of the GAN including the *generator* and the *discriminator*.

The representative architecture of the GAN used here is shown in Fig. 2. The neural networks of the discriminator and the generator are mainly composed of convolutional (Conv) and de-convolutional (Dconv) layers. The size of the input noise vector *z* to the generator is $m=50$ and its output is a 28-by-28 matrix comprised of elements valued between 0 and 1, representing the absence or presence of a metal film, respectively. Here, the generator NN is composed of a fully-connected (FC) layer that connects the 50-dimensional noise vector to $128\times 7\times 7$ neurons. This layer is followed by 2 deconvolution layers of 128 filters with (4, 4) kernel size and LeakyReLU activation function with $\alpha =0.2$. The discriminator NN is composed of 3 Conv layers of 64 filters with (3, 3) kernel size and the same activation function. The discriminator NN ends with a fully-connected layer that outputs one variable between 0 and 1 with a sigmoid activation function. Further details of the implemented networks are listed in Table 1.

**Table 1:** Neural Networks of the GAN in Fig. 2.

Layer	Specs	Output size
**Generator**		
Input		$m=50$
FC	Neurons = $128 \times 7\times 7$	$(128 \times 7\times 7)$
	activation: ‘LeakyReLU ($\alpha =0.2$)’	
Reshape		(7, 7, 128)
Dconv	$\#$ of filters = 128, kernel size = (4, 4)	
	Strides = (2, 2), padding =‘same’	(14, 14, 128)
	Activation: ‘LeakyReLU ($\alpha =0.2$)’	
Dconv	Same as above	(28, 28, 128)
	$\#$ of filters = 1, kernel size = (7, 7)	
Conv	Padding =‘same’, activation: ‘sigmoid’	(28, 28, 1)
**Discriminator**		
Input		(28, 28, 1)
Conv	$\#$ of filters = 64, kernel size = (3, 3)	
	Strides = (2, 2), padding =‘same’	
Conv	Activation: ‘LeakyReLU ($\alpha =0.2$, dropout rate = 0.4)	( , 14, 14, 64)
	Dropout rate = 0.4	
Conv	Same as above	(7, 7, 64)
Conv	Same as above	(4, 4, 64)
Flatten		$( , 4 \times 4 \times 64)$
FC	Neurons = $4 \times 4 \times 64$, activation = ‘sigmoid’	(, 1)

*FC* fully-connected layer, *Dconv* de-convolutional layer, *Conv* convolutional layer.

Here, the mere generation of different shapes for the scatterers of the polarizing met-atoms is not sufficient. Therefore, the GAN needs to be provided with the images of the scatterers of meta-atoms that meet specific set of electromagnetic properties. That way, the generator learns to produce meta-atoms that are compliant with certain electromagnetic constraints. Consequently, the training data is curated in two steps to include images of the scatterers that result in multi-band polarizers with orthogonal reflected CP waves between the adjacent bands.

**Figure 3:**
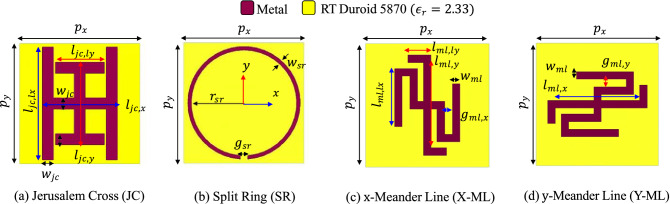
Primitives used for training the GAN: (**a**) Jerusalem cross, (**b**) split ring, (**c**) meander line in *x*-direction, and (**d**) meander line in *y*-direction. Ranges of each variable are defined in Table 2.

In the first curation step, specific shapes of scatterers, called primitives, here, instead of random pixelated scatterers are considered. These primitives shown in Fig. 3 chosen based on our design experience offer adequate degrees of freedom to provide meaningful scattering parameters in the frequency bands of interest. Through this step, it is possible to not only expedite the search for the optimized design, but to reduce the number of the required training samples compared to random pixelated shapes. The possible dimensions for the design variables of these scatterers are listed in Table 2. About 4700 random scatterers from these primitives are selected, placed on top of a grounded dielectric slab of RT Duroid 5870 ($\epsilon _r=2.33$) with 1.525 mm thickness, and is simulated in Ansys HFSS with Floquet port excitation and periodic boundary conditions under normal and oblique incidence at $30^\circ$. The linear-polarized reflection coefficients, $\Gamma _{xx}, \Gamma _{xy}, \Gamma _{yx}, \Gamma _{yy}$, from which $\Gamma _{xy}, \Gamma _{yx}$ are close to zero, are stored. In the second step, images of the scatterers that result in $e_{AR}<5.5$ for their meta-atoms plus their $90^\circ$-rotated versions are only included in the training data. It is worth mentioning that the upper limit of $e_{AR}=5.5$ corresponds to a single-band polarizer. Therefore, a training data sample should at least perform as a polarizer in a single band.

**Table 2:** Dimensions of the primitives in Fig. 3.

Primitive	Parameter	Value (mm)
JC	$l_{JC,x/y}$	[1.00:0.25:7.75]
	$l_{JC,lx/ly}$	[0.50:0.25:5.00]
	$w_{JC,x/y}$	[0.25:0.25:1.00]
SR	$r_{sr}$	[1.00:0.25:2.6]
	$g_{sr}$	[0.25:0.25:3.00]
	$w_{sr}$	[0.25:0.25:1.00]
X-ML & Y-ML	$l_{ml,x/y}$	[1.00:0.25:7.75]
	$l_{ml,lx/ly}$	[0.50:0.25:5.00]
	$g_{ml,lx/ly}$	[0.25:0.25:0.75]
	$w_{ml,lx/ly}$	0.50

The weights of the networks are optimized by the backpropagation^63^ of the gradients of the loss function in Eq. (4P) in an Adam optimizer with learning rate of $l_r=0.0001$ and parameter $\beta _1=0.9$. After 200 epochs with a batch size of 256, the discriminator was successful in predicting probability values close to 1 for $91\%$ of the training samples and probability values close to 0 for $89\%$ of the generated samples. Tensor Flow-backend Keras libraries in Python were employed to implement and train the machine learning models.

After training of the GAN is completed, the generator is integrated in a particle swarm optimization (PSO)^64^ step. The PSO is performed in the 50-dimensional noise vector space with *P* number of particles for *I* iterations where each particle of each swarm is fed into the generator and converted to an image of a scatterer. The position of the *j*th particle, $x_j$, is updated at the $(k+1)$th iteration based on^64^

5 $$\begin{aligned} {x_j(k+1) = x_j(k) + v_j(k+1)}, \end{aligned}$$

where

6 $$\begin{aligned} {x_j(k+1) = w \times v_{j,k}(m) + c_1 \times (p_j - x_j) + c_2 \times (p_g - x_j).} \end{aligned}$$

$p_j$ is the particle’s best position up till $(k+1)$th iteration and ${p_g}$ is the swarm’s best position. $c_1$ and $c_2$ are the cognitive and social parameters, respectively, that control the particle’s behavior to follow its personal best or the swarm’s global best position. Overall, this determines if the swarm is explorative or exploitative in nature. In addition, a parameter *w* controls the inertia of the swarm’s movement. The performance of the PSO is controlled by the choices of *P*, *I*, $c_1$, $c_2$, and *w*. Here, these parameters are empirically selected to obtain the best results. The PSO is implemented using PySwarms library^65^ in Python. The swarm’s direction is guided by the error value associated with the scatterer Eq. (2) obtained from the simulation results of the generated scatterer on top of the aforementioned grounded dielectric.

## Inverse design of dual and triple-band polarizers

Here, to assess the effectiveness of the proposed method to employ a GAN for designing a multi-band reflective polarizer, we design a dual-band as well as a triple-band example. For each polarizer, specific sets of $AR_{min}$ and $AR_{max}$ masks are defined based on the frequency bands of interest and $e_{AR}$ in Eq. (2) is used to find the optimized design. The optimization process is performed independently and separately for the dual-band and triple-band polarizers. However, the generation of the data set and training of the GAN are done only once and the same generator is employed for both inverse designs.

**Figure 4:**
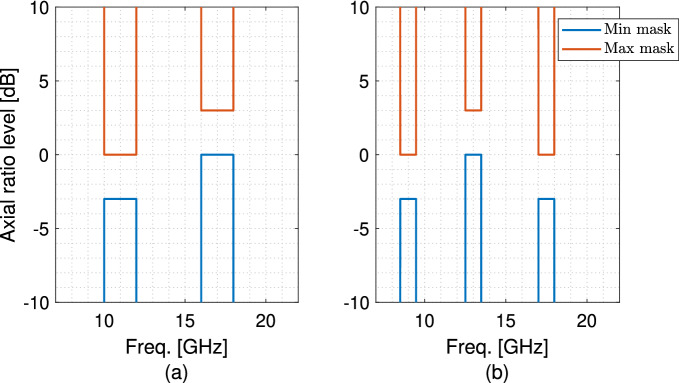
AR dispersive minimum and maximum masks for the (**a**) dual-band and (**b**) triple-band polarizers.

### Dual-band polarizing surface

The dual-band polarizer is designed to operate with 2.0 GHz and 1.5 GHz bandwidths with the center frequencies that are allowed to vary in $10.75-11.25$ GHz and $17.0-17.5$ GHz, respectively, while maintaining orthogonal CP polarizations between the two bands. The minimum and maximum masks are defined as shown in Fig. 4a. It is worth noting that in the lower band, the minimum and the maximum levels are indicated as $-3.0$ dB and 0.0 dB, respectively, to enforce the AR in this band to have a negative value, corresponding to an LHCP wave. Conversely, in the higher band, the minimum and the maximum levels are indicated as 0.0 dB and $+3.0$ dB, respectively, to enforce the AR in this band to have a positive value, corresponding to an RHCP wave. To accommodate the reverse, which can happen if the incident field or the scatterer is rotated $90^\circ$, the vertically-flipped version of this mask is also considered to enforce an RHCP wave in the lower frequency band and an LHCP wave in the higher frequency band. Then, $e_{AR}$ in Eq. (2) is calculated for both sets of the $AR_{min}$ and $AR_{max}$ masks and the minimum value is attributed to the meta-atom under test.

**Figure 5:**
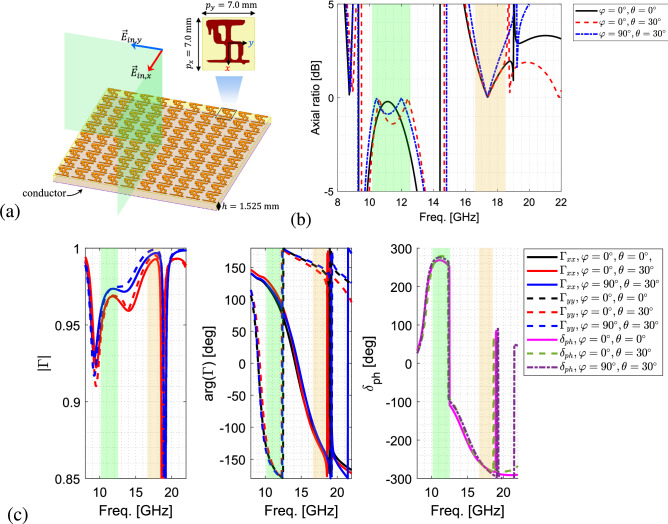
(**a**) The structure of optimized dual-band reflective polarizer. (**b**) The axial ratio, (**c**) the amplitude, phase and phase difference, $\delta _{ph}$, of the linear-polarized reflection coefficients $\Gamma _{xx}$ and $\Gamma _{yy}$ of the optimized dual-band polarizer for normal and oblique incidence at $\theta =30^\circ$ in both $\varphi =0^\circ$- and $\varphi =90^\circ$-planes. The negative dB values of AR represent the switch in the handedness of the CP wave.

The optimized dual-band polarizer is shown in Fig. 5a. As can be seen, the scatterer of this polarizer has a new generated shape bearing some resemblance to the Jerusalem cross and meander line primitives shown in Fig. 3. The axial ratio of the reflected CP wave by the optimized dual-band polarizer under normal and oblique incidence of $\theta =30^\circ$ in both $\varphi =0^\circ$- and $\varphi =90^\circ$-planes is shown in Fig. 5b. It is worth noting that exciting the surface by both *x*- and *y*-polarized electric fields simultaneously in the $\varphi =0^\circ$-plane, as shown in Fig. 5a, is equivalent to exciting the structure by a $45^\circ$-slanted electric field. The green ($10.15-12.55$ GHz) and orange ($16.60-18.50$ GHz) bars indicate the mutual frequency bands where the amplitude of the AR under both normal and the mentioned oblique incidence is less and equal to 3.0 dB. Therefore, with a frequency shift, the polarizer meets the bandwidth constraints, resulting in $21.2\%$ and $10.8\%$ bandwidths in the lower and higher frequency bands, respectively. It should be noted that the polarizer performance remains the same but with orthogonal polarizations in case it is excited by a $135^\circ$-slanted electric field.

The amplitude and phase of the linear-polarized reflection coefficients $\Gamma _{xx}$ and $\Gamma _{yy}$ of the optimized dual-band polarizer for normal and oblique incidence are shown in Fig. 5c, where the green and orange bars indicate the frequency bands in which the $|AR|\le 3.0$ dB. From Fig. 5c, it is evident that both $\Gamma _{xx}$ and $\Gamma _{yy}$ are equal and close to 1 with $+270^\circ$ and $-270^\circ$ in the lower and higher bands, respectively. It can also be seen that $\Gamma _{xx}$ and $\Gamma _{yy}$ stay stable when the incident wave shifts from normal to oblique angles equal to $\theta =30^\circ$ in both $\varphi =0^\circ$- and $\varphi =90^\circ$-planes. The amplitude drop at higher frequencies can be attributed to the propagation of the higher order modes linked to the scatterer spacing.

### Triple-band polarizing surface

After the successful design of a dual-band polarizer, we challenged the generator to synthesize a triple-band polarizer, where the CP wave is switched between the adjacent bands. The frequency bands of interest are $8.5-9.5$ GHz, $12.5-13.5$ GHz, and $17-18$ GHz, shown in Fig. 4b. To enforce orthogonality of the CP waves between the adjacent bands, the levels of the $AR_{min}$ in the bands of interest are $-3.0$ dB, 0.0 dB, and $-3.0$ dB, respectively. The levels of the $AR_{max}$ in the bands of interest are 0.0 dB, 3.0 dB, and 0.0 dB, respectively. Similarly, the vertically-flipped version of the triple-band set of $AR_{min}$ and $AR_{max}$ is also considered to find the optimized triple-band polarizer.

**Figure 6:**
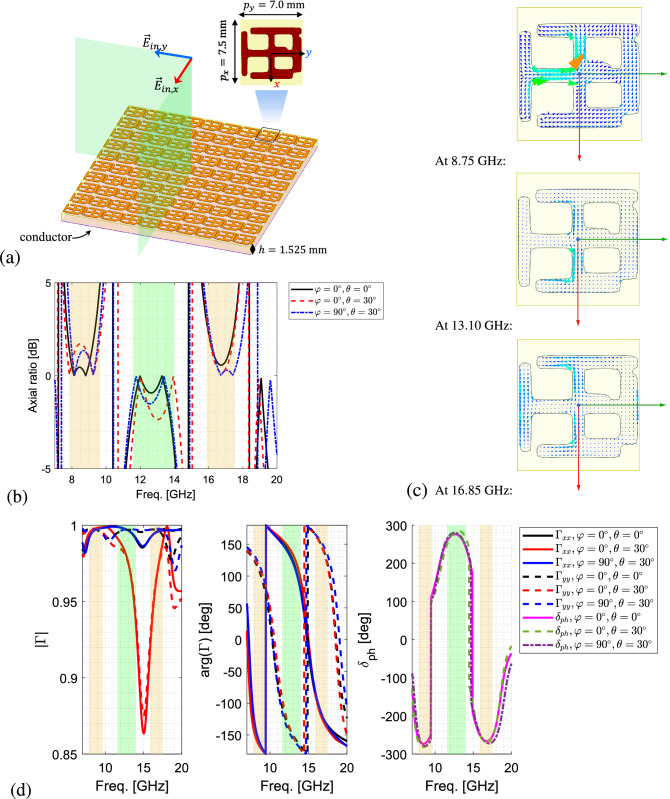
(**a**) The structure of optimized triple-band reflective polarizer. (**b**) The axial ratio of the optimized triple-band polarizer for normal and oblique incidence at $\theta =30^\circ$ in both $\varphi =0^\circ$- and $\varphi =90^\circ$-planes. The negative dB values represent the switch in the handedness of the CP wave. (**c**) The excited surface currents at 8.75, 13.10, and 16.85 GHz under normal incidence of *x*- and *y*-polarized electric fields. (**d**) The amplitude, phase and phase difference, $\delta _{ph}$, of the linear-polarized reflection coefficients $\Gamma _{xx}$ and $\Gamma _{yy}$ for normal and oblique incidence at $\theta =30^\circ$ in both $\varphi =0^\circ$- and $\varphi =90^\circ$-planes.

The optimized triple-band polarizer is shown in Fig. 6a. As it can be seen, the scatterer of this polarizer is similar to a Jerusalem cross shown in Fig. 3, but with a distinct property that the *x*- and *y*-directed loading are connected on the positive side of the *y*-axis. Moreover, another important feature that enables the triple-band operation is the wider *x*-directed loading on the positive side than the one on the negative side of *y*-axis. This is not a feature that was included in the training samples of the Jerusalem cross primitives. However, the generator has created it to meet the triple-band criteria.

The axial ratio of the reflected CP wave by the optimized triple-band polarizer under normal and oblique incidence of $\theta =30^\circ$ in both $\varphi =0^\circ$- and $\varphi =90^\circ$-planes is shown in Fig. 6b. It can be seen that in $7.85-9.40$ GHz, $11.58-14.0$ GHz, and $15.88-17.53$ GHz, the amplitude of the AR under both normal and the mentioned oblique incidence is less and equal to 3.0 dB. Therefore, the polarizer meets the bandwidth constraints, resulting in $20.6\%$, $18.8\%$, and $9.5\%$ bandwidths, respectively. It can also be seen that the sign of the AR between the adjacent bands switches as required. It should be noted that the polarizer performance remains the same but with orthogonal polarizations in case it is excited by a $135^\circ$-slanted electric field.

The excited surface currents at 8.75, 13.10, and 16.85 GHz when the surface is excited by normal incidence of *x*- and *y*-polarized electric fields are shown in Fig. 6c. It can be seen how each part of the scatterer plays a role at different frequencies to realize the required $(2i+1)\pi /2$ phase difference between the *x*- and *y*-polarized reflected electric fields, which yields to a CP reflected field. Shown in Fig. 6c, at 8.75 GHz, the two orthogonal centre parts of the scatterer are both excited but with different surface current densities which causes the difference between the phase of the reflected waves in *x*- and *y*-directions. At 13.10 GHz, it can be seen that the surface current densities are most excited along the *x*-directed feature at the center of the scatterer, suggesting manipulation of only *x*-directed fields. Finally, at 16.85 GHz, more current densities are excited along the *x*-directions not only at the center features but along the edges of the scatterer. From the lower to higher frequencies, it is evident that the difference between the excited current densities in *x*-direction and *y*-direction is growing to introduce more phase difference between the reflected fields in these directions and alternate the polarization.

The amplitude and phase of the linear-polarized reflection coefficients $\Gamma _{xx}$ and $\Gamma _{yy}$ of the optimized triple-band polarizer for normal and oblique incidence are shown in Fig. 6d, where the green and orange bars indicate the frequency bands in which the $|AR|\le 3.0$ dB. From Fig. 6d, it is evident that both $\Gamma _{xx}$ and $\Gamma _{yy}$ are equal and close to 1 with $-270^\circ$, $+270^\circ$, and $-270^\circ$ in the bands, respectively. It can also be seen that $\Gamma _{xx}$ and $\Gamma _{yy}$ remain stable when the incident wave moves from normal to oblique angles, corresponding to $\theta =30^\circ$ in both $\varphi =0^\circ$- and $\varphi =90^\circ$-planes.

## Experimental verification of the triple-band polarizer

To demonstrate the feasibility of the inverse designed polarizers here, we fabricated and experimentally validated a prototype of the triple-band polarizer under both normal and oblique incidence at $\theta =30^\circ$ in $\varphi =0^\circ$- and $\varphi =90^\circ$-planes. A 254 mm $\times 406.4$ mm prototype of the polarizer that includes $36\times 58$ meta-atoms is fabricated. The scatterers are etched on a 1.578-mm thick RT Duroid 5870 with $18 \upmu$m-copper cladding.

**Figure 7:**
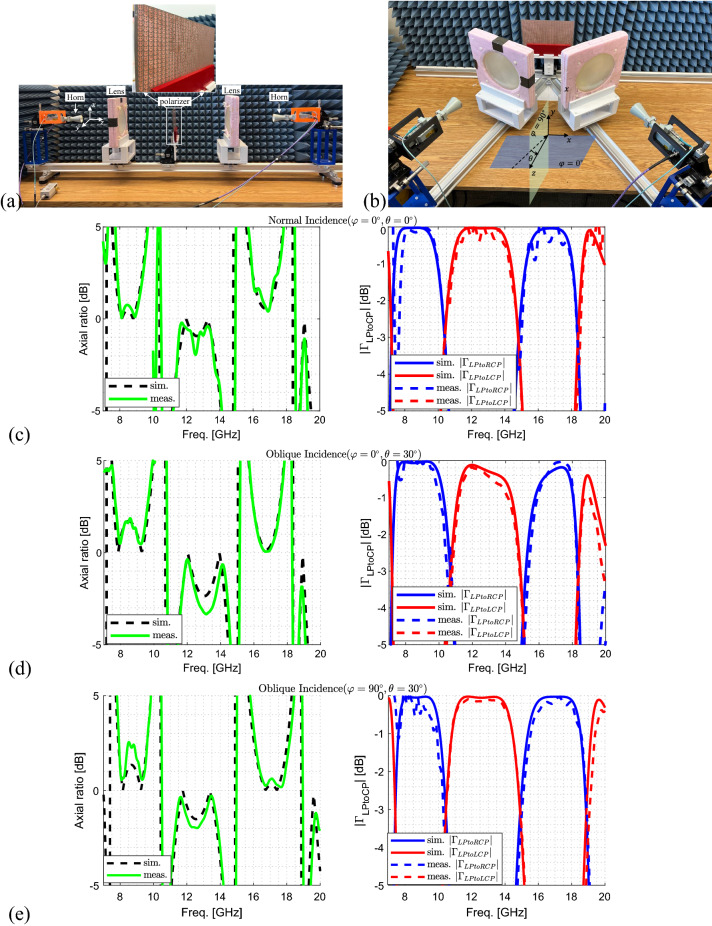
Quasi-optical setup for measuring reflection coefficients of the prototype under (**a**) normal incidence and (**b**) oblique incidence. The measured and simulated axial ratio and the LP-to-CP reflection coefficients of the optimized triple-band polarizer for (**c**) normal, and oblique incidence at (**d**) $\theta =30^\circ$ in $\varphi =0^\circ$-plane and (**e**) $\theta =30^\circ$ in $\varphi =90^\circ$-plane.

The linearly-polarized reflection coefficients of the prototype under normal incidence are measured using the quasi-optical setup shown in Fig. 7a. This setup consists of two identical symmetrically-positioned transmitting (Tx) and receiving (Rx) sides composed of a horn antenna as the radiating element and a lens. On each side, the spherical wave of the horn antenna is transformed by the biconvex lens to a Gaussian beam with the waist diameter of 90.0 mm. The sample under test is placed between the lenses at this waist where the phase profile is planar. The optimal distance between the horn antennas and lenses is determined for this transformation to be 150 mm and 200 mm for the X- and Ku-band horns^66,67^, Appendix C]. The distance between each lens and the sample under test is 300 mm fixed. The prototype is characterized in the frequency ranges 7.0-10.0 GHz and 10.0-20.0 GHz using X-band and Ku-band horns, respectively.

To characterize only the polarizer using this setup, first, a thru-reflect-line (TRL) calibration is performed. In this three-step process, the transmission and reflection coefficients at the ports of the two horns are measured when: (1) the sample is absent (thru); (2) a flat sheet of copper is under test (reflect); and (3) one of the horns plus the lens before it are moved for a distance of free-space quarter-wavelength at the center frequency of the band (line). After the calibration is completed, the signal attenuation and phase delay due to free-space propagation are determined and excluded from the sample measurements. Moreover, to exclude the multiple-reflections between the horns and lenses, time-gating is also employed.

The setup shown in Fig. 7b is employed to measure the reflection coefficients of the prototype under oblique incidence at $\theta =30^\circ$ in the $\varphi =0^\circ$-plane. The prototype is then rotated by $90^\circ$ to measure the reflection coefficients in $\varphi =90^\circ$-plane under $30^\circ$-oblique incidence. Here, since no thru measurement can be defined between the two horns when the sample is absent, first, a standard calibration is performed on the cables using an electronic calibration kit to exclude cable losses from the measurements. Then, the reflect step of the TRL calibration is performed manually where the results of the sample are referenced to the ones of a flat sheet of copper. After confirming that the cross-polarization reflection coefficients are negligible in these measurements, the axial ratio of the reflected waves are calculated using Eq. 1 for both normal and oblique incidence.

The measured axial ratios are summarized and compared with simulation results in Fig. 7c–e. As can be seen, there is a good agreement between them for normal and oblique incidence, with the axial ratio remaining below 3.0 dB maximum level in all the desired bands. The major difference occurs for $\theta =30^\circ$ incidence in $\varphi =0^\circ$-plane, shown in Fig. 7d, at 13.10 GHz. The simulated axial ratio has the maximum value of 2.3 dB in the $11.6-14.45$ GHz-band while the measured axial ratio becomes 3.3 dB, marginally exceeding the specified 3.0 dB maximum level. This error can be attributed to inevitable fabrication and measurement inaccuracies. Finally, the simulated and measured LP-to-CP reflection coefficients in dB are calculated based on

7 $$\begin{aligned} |\Gamma _{LPtoCP}| = 20\log _{10}|[\frac{1}{2}(\Gamma _{xx}\pm j\Gamma _{yy})]| \end{aligned}$$

and compared in Fig. 7c–e. Besides the good match between the measured and simulated results, it can be seen that an LP wave is indeed converted to orthogonal CP waves in adjacent bands.

## Conclusion

An end-to-end approach employing a generative adversarial network (GAN) was used to design single-layer reflective polarizers with complex dispersive electromagnetic properties. These requirements included dual and triple broadband linear-to-circular polarization conversions with orthogonal polarizations produced in adjacent bands not only for normal incident waves but also oblique incidence up to $\theta =30^\circ$. GANs typically require a large training data set, however, using data curation and augmentation, the required simulations to prepare the training data were efficiently reduced. Moreover, once the GAN training was completed, new designs with new sets of requirements were quick to produce.

The feasibility of the optimum designs by the proposed approach was validated through prototyping and measurement, where good agreement with the simulated results was obtained. The results confirmed that the implemented GAN is sufficiently powerful in exploiting the limited information provided in a relatively small training data set to generalize/ extrapolate desired features, create new geometries, and explore the solution space of the EM surfaces. These capabilities can be leveraged to derive a sophisticated and dispersion-optimized design. As for the future directions, surrogate models to predict the electromagnetic properties of the generated meta-atoms can greatly expedite the optimization process. Moreover, other design parameters such as the substrate permittivity and thickness can be added as degrees of freedom to improve the results further.

## Methods

### Simulation of the unit cell

Full-wave electromagnetic simulations are performed using the Ansoft High Frequency Structure Simulator (HFSS) commercial software. A single unit cell is simulated in a three dimensional environment by assigning Floquet ports on its top and bottom surfaces for normal and oblique incident plane-wave excitation and terminating its sides by periodic boundary conditions to simulate an infinite array.

### Machine learning model developments

The implementation and training of the machine learning models are done using TensorFlow-backend Keras libraries in Python.

## Data Availability

The datasets used and/or analysed during the current study available from the corresponding author on reasonable request.
